# Health Service‐Related Determinants of Health‐Seeking Behavior during Malaria Fever in a High‐Risk Region of Nepal

**DOI:** 10.1002/hsr2.70739

**Published:** 2025-04-21

**Authors:** Ashok Kumar Paudel, Muni Raj Chhetri, Prem Prasad Panta, Nibha Rani Pandey

**Affiliations:** ^1^ Department of Research and Development, National Open College Pokhara University Nepal; ^2^ Department of Public Health, National Open College Pokhara University Nepal; ^3^ KIST Medical College & TH, TU Nepal; ^4^ RKDF University Ranchi India

**Keywords:** health service, health‐seeking behavior, high‐risk area, malaria fever, Nepal

## Abstract

**Background and Aims:**

Malaria continues to be a significant global health challenge, leading to preventable illnesses and loss of lives. This cross‐sectional descriptive study, employing a mixed‐method approach, aimed to investigate the health service‐related factors affecting health‐seeking behavior during episodes of malaria fever in a high‐risk region of Nepal.

**Methods:**

Using a cross‐sectional descriptive design with a mixed‐method approach, the research focused on Kanchanpur district, selected from 20 high‐risk malaria districts of Nepal. Belauri Municipality within Kanchanpur, identified for its concentration of high and moderate‐risk wards, was the specific study area. A random selection process identified 387 households for a comprehensive survey. Face‐to‐face interviews with household heads were conducted after obtaining written informed consent and ethical approval from the Nepal Health Research Council. Data analysis, employing statistical measures such as percentages, frequency, mean, and the Chi‐square test, was performed using SPSS version 20.

**Results:**

Means of reaching the public health facility (AOR = 8.324, 95% CI = 3.677–18.844, *p* < 0.001), time to reach public health facility (AOR = 0.234, 95% CI = 0.059–0.918, *p* = 0.017), regular availability of service providers (AOR = 0.155, 95% CI = 0.054–0.442, *p* < 0.001), most of the time availability of service provider (AOR = 0.115, 95% CI = 0.039–0.334, *p* < 0.001), opening hours of health facility (AOR = 0.301, 95% CI = 0.156–0.581, *p* < 0.001), and perceived quality of service (AOR = 0.256, 95% CI = 0.154–0.424, *p* < 0.001), having to pay for transportation (COR = 0.275, 95% CI = 0.164–0.461, *p* < 0.001), travel cost (COR = 0.744, 95% CI = 0.640–0.865, *p* < 0.001), were the significant factors in health‐seeking behavior during malaria fever.

**Conclusion:**

Collectively, these findings provide a comprehensive understanding of the multifaceted nature of health‐seeking behavior in the context of malaria fever and emphasize the importance of addressing various factors to improve healthcare utilization. Subsidized transportation for the poor helps to overcome financial barriers, establish additional facilities or mobile clinics to reduce travel time, ensure healthcare workforce availability, extend health facility hours, patient‐centered initiatives to enhance service quality can increase the likelihood of people seeking care from modern health facilities in Nepal.

## 1 Introduction

Globally, the number of malaria cases rose to 247 million in 2021, up from 245 million the previous year. Meanwhile, the estimated number of deaths due to malaria declined to 619,000 in 2021, compared to 625,000 in 2020. Despite the reduction in deaths, malaria continues to pose a significant challenge to global health and results in both preventable illnesses and fatalities [1]. Early diagnosis and treatment of malaria both at health facilities and household levels, are the strategies developed by the World Health Organization (WHO) to reduce the morbidity and mortality burden of malaria [2]. The Global Technical Strategy for Malaria 2016‐2030 aims to reduce the malaria burden by 90% by 2030 [3, 4]. The recent study done in Nepal (2018) concluded that 67 out of the 77 districts of Nepal and about 43.26% of the total population are residing in areas at risk (high, moderate, and low) of malaria. The study further streamlined that 202 wards in 20 districts (as per the new federal structure) are at high or moderate risk of malaria. In Nepal, approximately 3.96% of the total population are living in malaria endemic (high & moderate risk) areas, and 0.22 million live‐in high‐risk areas (49 wards), 0.93 million in moderate risk areas (253 wards), and 11.34 million in low risk areas (2,543 wards) [5]. So, this cross‐sectional descriptive study, employing a mixed‐method approach, was conducted to assess the health service‐related factors influencing health‐seeking behavior during suspected malaria fever in high‐risk wards of Belauri Municipality, Nepal.

## 2 Research Question

What are the health service‐related factors that have influence on seeking healthcare during suspected cases of malaria fever in high‐risk areas of Nepal?

## 3 Literature Review

In the changed federal context of Nepal, local‐level health facilities are basic implementation units to deliver basic health care services (BHCS) including prevention and treatment of malaria [6]. Besides the formal health care system under the Ministry of Health and Population (MoHP) and private hospitals/nursing homes/clinics, traditional forms of healing are also widely prevalent in Nepal and are generally practiced by the local healers/traditional faith healers known as Dhami/Jhakries. Many people in rural areas still believe in Dhami/Jhakris as treatment for illnesses, as faith healing remains a prominent healthcare practice in Nepal [7].

### 3.1 Illness, Sickness and Disease

Illness is the subjective experience of “not feeling well,” offering an individual's personal perspective [8]. On the other hand, sickness is a broader concept that involves perceiving a disorder in a more general sense across a population, taking into account macro‐social factors like economic, political, and institutional influences [9, 10]. Disease, as perceived by healthcare practitioners and modern healers, is what they are trained to recognize through their biomedically oriented perspectives. It refers to the malfunctioning of biological and/or psychological processes within an individual's body [11]. These terms represent distinct facets of the health experience, ranging from the individual's feelings of unwellness to the societal recognition of illness and the biomedical understanding of disease.

### 3.2 Health Seeking Behavior as Different Perspective

Health‐seeking behavior encompasses any actions taken by individuals who believe they are unwell and are seeking an appropriate remedy [12, 13, 14]. Tipping and Segall (1995) distinguish health seeking behavior between two broad types‐ Firstly, it emphasizes the “end point” (utilization of the formal health care system, or health care seeking behavior). Such end points of the decision making process or utilization of a formal health care system are normally recorded in attendance at the health facility. Next, are those which emphasize the ‘process’ (illness response or health‐seeking behavior) [15, 16].

#### 3.2.1 Health Care Seeking Behaviors or Utilization of the Formal Health Care System

The decision to engage with a particular source of treatment is influenced by a variety of socio‐demographic aspects, access to services and perceived quality of the service. The first trend involves determinants that act as intermediaries between patients and healthcare services. Next, generally fall into geographical, social, economic, cultural, and organizational factors [15].

#### 3.2.2 Health Seeking Behavior or the Process of Illness Response

Health‐seeking behavior, also known as the illness response process, is deeply rooted in psychology and focuses on the broader spectrum of health‐seeking behaviors. The process of illness response investigates the process of health care seeking. Such a process may include identification of pathways to the formal health care system, often commencing with home care and traditional healers and extending to the formal system, and pathways may differ according to the presenting condition [16].

Therefore, for this study we have made use of the concept ‘health seeking behavior’ in the meaning and terms “Health‐seeking behavior can be defined as any activity undertaken by individuals who perceive themselves to have a health problem or to be ill for the purpose of finding an appropriate remedy” which includes both medical and nonmedical/traditional resources in this process [14].

## 4 Theoretical and Conceptual Framework

Andersen's behavior model has been adopted for the study. Andersen's early versions of the model, first introduced in 1973, marked a pivotal moment in the development of his groundbreaking concept. Andersen presented a new version of his model that supports need, predisposing, and enabling factors [17, 18, 19, 20]. Factors influencing the need for health services encompass specific illnesses, conditions, and individual health statuses. Predisposing factors include demographic characteristics, social structural variables, as well as an individual's underlying beliefs, attitudes, and knowledge related to health services. Enabling factors revolve around the availability of resources, whether at the individual level or within the community [17, 18, 19, 20, 21].

Andersen's Behavioral Model of Health Services Use is widely recognized as a comprehensive framework for understanding and predicting health‐seeking behavior. Its adoption in this study underscores its utility in exploring the multifaceted factors influencing healthcare utilization. The model has been successfully applied in diverse settings and populations to examine health‐seeking behaviors related to chronic diseases, preventive care, and acute illnesses. It is particularly relevant in low‐ and middle‐income countries (LMICs) like Nepal, where social, economic, and cultural factors significantly impact healthcare access. By identifying barriers and facilitators to healthcare access, the model aids policymakers in designing targeted interventions. For example, understanding enabling factors can guide resource allocation to underserved areas, while addressing predisposing factors can inform educational campaigns.

**Figure 1:**
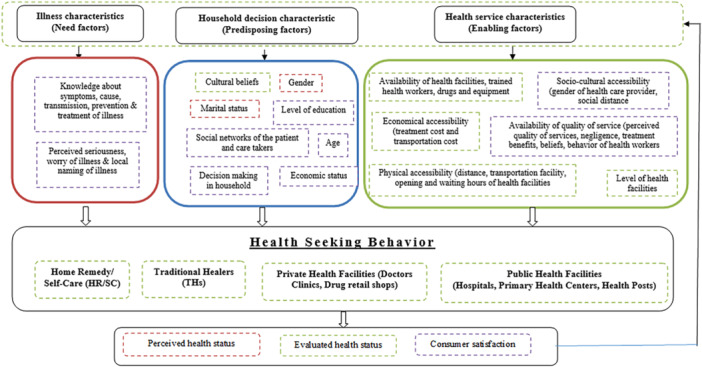
Conceptual framework based on Andersen's (1973) Behavioral Model of Health Services Use.

Andersen's model provides a robust and adaptable framework for analyzing healthcare utilization, particularly in settings like malaria‐prone areas of Nepal. However, its limitations—such as its static nature, insufficient focus on cultural and systemic barriers, and challenges in contextual application—should be acknowledged and addressed. Complementing the model with additional frameworks or localized adaptations can enhance its applicability and effectiveness in understanding health‐seeking behaviors in diverse populations. See Figure 1 for conceptual framework based on Andersen's (1973) Behavioral Model of Health Services Use employed in this study.

## 5 Materials and Methods

### 5.1 Study Design and Setting

In this study, a cross‐sectional descriptive design with a concurrent triangulation mixed‐method approach was implemented. Both qualitative and quantitative data were simultaneously collected and analyzed to compare and contrast the results, thereby validating the findings in wards of Belauri municipality of Nepal, from 5th March to 20th May 2023. Nepal is divided into 77 districts; districts comprise municipalities, and municipalities are divided into wards. The Malaria Micro‐Stratification report of Epidemiology and Disease Control Division/Ministry of Health and Population/Nepal (2018) found that 20 districts in Nepal have moderate and high risk of malaria. Among the 20 districts in Nepal with malaria risk, Kailali and Kanchanpur districts stood out with higher numbers of high and moderate‐risk wards (58 in Kailali and 43 in Kanchanpur). Using a simple random sampling method, Kanchanpur district was chosen. Within Kanchanpur district, Belauri Municipality, housing the highest numbers of high and moderate‐risk wards for malaria, was selected. Ward number 1, the sole high‐risk ward in Belauri, was chosen for the study. Additionally, 50% of the 8 moderate‐risk wards were randomly selected, totaling 5 wards (1, 2, 3, 4, and 6). Comprehensive household details for Belauri Municipality and the selected wards were prepared.

### 5.2 Study Population

As per the Municipal profile of Belauri Municipality in 2018, the selected wards (1, 2, 3, 4, and 6) comprised a study population of 4,637 households with a total population of 24,852. All households of selected wards were the study population for household survey.

### 5.3 Sample Size and Sampling Process for Household Survey

According to the profile of Belauri municipality, in 2018 there were 9970 total households. Household details of Belauri municipality and details of each selected ward (1, 2, 3, 4, and 6) of Belauri municipality were prepared. Selected wards (1, 2, 3, 4, and 6) of Belauri Municipality have 4637 households. These households of selected wards were the sampling unit. The number of household samples required for the household survey was determined by using formula (*n*) = N/(1 + Ne2). A total of 387 households were included for this study. The probability proportional to size (PPS) method was used to identify the sample size of each selected ward, and desired numbers of households from each Ward were identified by using the Simple Random Sampling (SRS) method.

### 5.4 Data Collection Tools and Techniques

Quantitative data were collected through a household survey, using a structured interview questionnaire with heads of households serving as respondents. Qualitative data were collected through In‐depth Interviews, Key Informant Interview and Focus Group Discussion methods. Individuals diagnosed with malaria infection during the last 1‐year period were purposively chosen as respondents for In‐depth Interviews (IDIs). Participants with malaria infection diagnosed by local health facilities were deliberately selected based on the records of those facilities. Two IDIs were conducted in each ward, totaling 10 IDIs to conclude the study. Health care providers and managers, teachers, social workers, community leaders, Female Community Health Volunteers (FCHVs), and traditional healers (TH) were respondents for Key Informant Interviews (KIIs). A total of 10 KIIs were conducted to complete the study. Men and women above 18 years old, residing within selected wards, participated in Focus Group Discussions (FGDs). Eight to ten people meeting the study population criteria were purposively selected for each FGD. Two FGDs were conducted in each ward, resulting in a total of 10 FGDs among different social and ethnic groups.

Open‐ended guiding questions were developed and presented to the respondents for the IDI and KII methods of data collection. A list of open questions or issues was developed to gather information through FGD techniques. For completeness, the collected data underwent editing, review, and checks.

Before data collection, respondents were assured that their responses would remain confidential and would not have any personal consequences. Face‐to‐face interviews were conducted in private settings to ensure that participants felt comfortable speaking openly. A structured interview guide was used to maintain consistency across respondents, and interviewers remained neutral, avoiding any indications of agreement or disagreement through facial expressions, gestures, or tone of voice. These measures were taken to minimize potential biases.

### 5.5 Data Analysis

Statistical Package for Social Sciences (SPSS) version 20 was employed to analyze the survey data. For the data generated from the household survey, percent distribution was used for descriptive analysis, and comparisons were made using the Chi‐square test to assess associations between dependent and independent variables.

Qualitative data from In‐depth Interviews (IDIs), Key Informant Interviews (KIIs), and Focus Group Discussions (FGDs) were analyzed manually. Initially, the qualitative data was organized using a thematic analysis approach. Key issues and themes were identified, and the responses to questions within these themes were grouped and summarized in a data analysis framework. Quotations that illustrated the views of the majority of participants or those in contradiction with the majority were extracted. These issues were then summarized by wards and health facility levels and finally integrated into the relevant sections. The data was further summarized by using all the original texts, listing all conceptual categories and patterns, and placing relevant information under these conceptual categories. Relationships were identified between the categories.

The qualitative data analysis process for this study was conducted systematically to derive meaningful insights from the information collected through In‐depth Interviews (IDIs), Key Informant Interviews (KIIs), and Focus Group Discussions (FGDs). The qualitative data were manually analyzed using a thematic analysis approach. Key issues and themes were identified based on the objectives of the study and the responses from participants. These themes served as the foundation for organizing and summarizing the data. Responses within each theme were grouped and summarized in a structured framework to ensure systematic organization of findings. Thematic responses were summarized by identifying recurring patterns and variations. Participant quotations that either reflected the consensus or contradicted the majority opinion were extracted to illustrate the breadth of perspectives. Data was summarized at multiple levels, including wards and health facilities, to capture location‐specific variations in perspectives. All original textual data were reviewed to identify conceptual categories and patterns. Relevant information was categorized under these conceptual themes, ensuring comprehensive coverage of the data. Relationships and connections between the identified conceptual categories were explored to understand underlying dynamics and contextual factors. This step aimed to link individual responses to broader themes and identify any patterns or trends across different groups and settings. The summarized data were integrated into relevant sections of the study report to provide a cohesive narrative. The qualitative findings were compared with quantitative data to validate and enrich the overall conclusions. Thematic analysis and categorization were reviewed iteratively to ensure accuracy and completeness. Key findings were cross‐checked with participants or stakeholders where possible to ensure credibility and trustworthiness.

This comprehensive process ensured that the qualitative data were analyzed rigorously, capturing diverse perspectives and providing a nuanced understanding of the research problem. The manual approach facilitated an in‐depth exploration of relationships and themes, enhancing the study's overall validity and reliability.

### 5.6 Validity and Reliability

Random sampling techniques were used to ensure unbiased representation of the total study population. External validity was addressed by reviewing relevant literature, and meeting, discussion were done with experts. Data collection tools were reviewed, translated into the Nepali language without losing the essence of questions, and pre‐tested in a similar setting before finalization. The local language was used to minimize bias, and the researchers established a purposeful rapport with participants to encourage open and honest responses. Written informed consent was also obtained from all participants, providing them with the right to withdraw from the study at any point without giving an explanation.

### 5.7 Ethical Approval and Informed Consent

Before data collection, the study's purpose was explained to the respondents, and written informed consent was obtained. Ethical approval was secured from the Nepal Health Research Council (NHRC) in Nepal.

## 6 Results

### 6.1 Sample Characteristics of the Survey Respondents

Table 1 provides information regarding socio‐demographic variables. The study reveals that the ages distribution is skewed towards the younger age ranges, with more than half (53.5%) of respondents falling in the 30–50 age range. About 70.5% of the respondents being male. Most respondents reported marital status as currently married (87.6%). A large majority of respondents were Hindu (89.4%). The largest number of respondents identify as Dalit (32.8%), and 69% of the respondents belong to nuclear families.

**Table 1:** Socio‐demographic information (*N *= 387).

Variables	Category	Frequency	Percent
Age (Mean = 41.33 yrs; SD = 12.7 yrs)	20–30	85	22.0
	30–40	110	28.4
	40–50	97	25.1
	50–60	52	13.4
	60 and above	43	11.1
Sex	Male	273	70.5
	Female	114	29.5
Marital status	Currently Married	339	87.6
	Divorced	8	2.1
	Single/Never married	12	3.1
	Widow/Widower	28	7.2
Religion	Hindu	346	89.4
	Buddhist	12	3.1
	Muslim	2	0.5
	Christian	27	7.0
Caste/ethnicity	Bhrahmin	14	3.6
	Chhetri	109	28.2
	Tamang	12	3.1
	Dalit	127	32.8
	Janajati	53	13.7
	Tharu	72	18.6
Family type	Nuclear	267	69.0
	Joint	120	31.0

### 6.2 Bivariate Analysis of Health Service‐Related Factors Associated With Health Seeking Behavior

Chi‐square analysis was performed to identify the association between dependent and independent variables. The level of significance has been set to 0.05 or 5% to measure the association between the variables. For this purpose, dependent variables have been grouped as‐ Home remedy/self‐care (HR/SC), Public health facility (HF), Private health facility (HF), and Traditional healer (TH).

Table 2 shows public hospitals (45.6%) as the nearest health facility. The most common means of reaching the public health facility was by foot (62.1%) and the usual time to reach the nearest public health facility was less than 15 min (33.8%). The chi‐square test shows that these variables are significant in determining the choice of health‐seeking behavior (*p* = < 0.05).

**Table 2:** Association between access to health facility and health seeking behavior (*N* = 387).

Variables	Category	HR/SC (%)	Public HF (%)	Pvt. HF (%)	TH (%)	Total (%)	Chi square	*p* value*
Nearest public health facility from house	Public hospitals	16.6	45.6	33.2	4.6	62.3	27.627	0.01
	PHCC	21.1	35.1	31.6	12.3	14.7		
	HP	10.8	67.5	14.5	7.2	21.4		
	Others	0.0	33.3	66.7	0.0	1.6		
Most common means to reach at public health facility	By foot	13.8	62.1	20.7	3.4	30.0	145.096	< 0.01
	Bicycle	26.5	28.6	4.1	40.8	12.6		
	Motor‐bike	13.2	49.1	37.7	0.0	27.4		
	Public vehicle	15.5	43.1	41.4	0.0	30.0		
Usual time to reach nearest public health facility	< 15 min	18.5	33.8	44.6	3.1	33.6	83.9	< 0.01
	15–30 min	14.6	58.6	23.3	3.5	51.1		
	30–45 min	20.7	34.5	6.9	37.9	7.5		
	45–60 min	6.7	60.0	26.6	6.7	7.8		

* *p* value significance at < 0.05.

#### 6.2.1 Theme: Distance of Health Facility and Transportation

FGD and KII findings suggested that in this low‐resource setting, people lack access to money and transportation that could quickly facilitate a higher level of care for their affected children; lack of funds prohibits timely escalation of care. Lack of money to finance the costs of transportation and allopathic care may influence the decision to seek care from local, traditional healers or informal private clinics rather than the government health centers located away.

“We have a truth in our community that the rich do not go to jail, the poor do not go to the hospital”

(Male, 52 yrs FGD)

“We get dizziness, leg pain, and bone pain while going to the health post by foot, and we become tired after returning”

(Female 63 yrs/FGD)

“To reach a health facility services for instance, some must walk, some use bicycles. With the introduction of motorcycle, taxi seems at least to serve us in seeking health care”

(FCHV, 39 yrs/KII)

“I hope the roads will also be improved. When it is raining, people die because they are not sent to the hospital in time”

(Female, 43 yrs/FGD)

“We are very poor and it is challenging to cover the cost of transportation to access healthcare facilities. If there were provisions for free or subsidized transportation, it would greatly assist us”

(Female, 42 yrs/FGD)

The Table 3 shows the association between usual travel‐cost to reach the nearest public health facility and health‐seeking behavior. Among the total sample of 387 respondents, 175 (45.2%) respondents had to pay for transportation to reach the nearest public health facility. The result also shows that among the 175 respondents who had to pay for transportation, 50 (28.6%) respondents reported that their usual travel cost was less than 60 rupees and 125 (71.4%) respondents reported that their usual travel cost was 60 rupees or above. The Chi‐square test shows that there is a significant association between usual travel‐cost and health‐seeking behavior (*p*‐value = < 0.05).

**Table 3:** Association between usual travel cost to reach public health facility and health seeking behavior.

Variables	Category	HR/SC (%)	Public HF (%)	Pvt. HF (%)	TH (%)	Total (%)	Chi square	*p* value*
Have to pay for transportation (*n* = 387)	Yes	22.3	42.3	24.0	11.4	45.2	28.533	< 0.01
	No	10.4	53.8	34.0	1.8	54.8		
Usual travel cost to reach nearest public health facility in NRs (*n* = 175)	< 60 rupees	28.0	36.0	16.0	20.0	28.6	8.046	0.04
	60 and above rupees	20.0	44.8	27.2	8.0	71.4		

* *p* value significance at < 0.05

The results presented in Table 4 suggest that the variables like‐ availability of service providers in the nearest public health facility, sufficient time to put complaints with service providers, waiting time, and opening hours of health facilities were statistically highly significant predictors of health‐seeking behavior (*p*‐value < 0.01). In addition, the perceived skill of the service provider to treat malaria was also a statistically significant predictor of health‐seeking behavior (*p*‐value < 0.05).

**Table 4:** Association between availability, skill of service provider in health facility and health seeking behavior.

Variables	Category	HR/SC (%)	Public HF (%)	Pvt. HF (%)	TH (%)	Total (%)	Chi square	*p* value*
Availability of service providers in nearest public HF (*n* = 387)	Regular available	19.3	46.9	30.7	3.1	49.6	48.826	< 0.01
	Most of time available	6.9	54.3	30.6	8.2	44.7		
	Most of the time unavailable	54.5	18.2	9.1	18.2	5.7		
Perceived skill of service provider to treat malaria in nearest public HF (*n* = 387)	Fully skilled	16.1	47.9	28.2	7.8	56.1	21.343	0.01
	Skilled	17.1	44.7	34.9	3.3	39.3		
	Poorly skilled	0.0	85.7	0.0	14.3	3.6		
	Not skilled	0.0	100.0	0.0	0.0	1.0		
Get sufficient time to put complaints with service providers (*n* = 387)	Yes, get sufficient time	14.6	48.5	32.9	4.0	83.2	23.941	< 0.01
	Don't get sufficient time	21.5	49.3	12.3	16.9	16.8		
Waiting time (in minute) for consultation after registration in public HF (*n* = 322)	5–10 min	20.5	35.9	32.1	11.5	24.2	31.6	< 0.01
	10–15 min	15.3	52.3	32.4	0.0	54.7		
	15–20 min	6.1	54.5	33.3	6.1	20.5		
	20–25 min	0.0	0.0	100.0	0.0	0.6		
Opening hours of PHF (*n* = 387)	Convenience	13.6	50.8	31.1	4.5	85.5	21.5	< 0.01
	Not convenience	28.6	35.7	19.6	16.1	14.5		

* *p* value significance at < 0.05.

#### 6.2.2 Theme: Availability and Skill of Service Providers

KII and IDI findings also suggested that the availability of skilled service providers in this study area also influences on health‐seeking behavior. Respondents describe experiences at government facilities where health‐care providers were not present, crucial medications were out of stock, and diagnostic testing was not performed.

“Our health centers are said to be open from 10 to 4 pm. Hardly, it opens for 3‐4 h and service providers usually give service only for 3‐4 h”

(Female, 32 yrs IDI)

“Most service providers working in this health center have their own private clinics and they spend more time there. They come late and go early from health centers”

(Community leader 56 yrs/KII)

The result of Table 5 shows that the majority of respondents (89.7%) reported that service providers had friendly behavior. However, this did not have a significant effect on health‐seeking behavior (*p*‐value of 0.202). The majority of respondents (76.2%) reported that they did not have difficulty understanding the advice or words of service providers and a statistically highly significant association between having difficulty to understand the advices/words of the service provider and health‐seeking behavior (*p*‐value = < 0.01) has been observed.

**Table 5:** Association between behavior of service provider, communication and health seeking behavior (N = 387).

Variables	Category	HR/SC (%)	Public HF (%)	Pvt. HF (%)	TH (%)	Total (%)	Chi square	*p* value*
Behavior of service provider	Friendly	16.5	47.8	28.8	6.9	89.7	8.533	0.20
	Not friendly	11.1	50.0	38.9	0.0	9.3		
	No comment	0.0	100.0	0.0	0.0	1.0		
Across with difficulty to understand the advices/words of service provider	Yes	26.1	28.3	33.6	12.0	23.8	25.6	< 0.01
	No	12.5	54.9	28.2	4.4	76.2		

* *p* value significance at < 0.05.

Table 6 presents the association between various factors (drug sufficiency, diagnostic facilities, perceived quality of service) and health‐seeking behavior for the treatment of malaria. The results of the chi‐square test show that there is a statistically highly significant association between the independent variables like‐ sufficiency of drugs, provision of microscopic examination for malaria confirmation, and perceived quality of services available and health‐seeking behavior (*p*‐value < 0.01). Overall, the result suggests that the availability and sufficiency of drugs, diagnostic facilities, and perceived quality of service play a significant role in determining the health‐seeking behavior of individuals for the treatment of malaria.

**Table 6:** Association between sufficiency of drug, diagnostic facilities, perceived quality of service and health seeking behavior (*N* = 387).

Variables	Category	HR/SC (%)	Public HF (%)	Pvt. HF (%)	TH (%)	Total (%)	Chi square	*p* value*
Sufficiency of drugs	Sufficient	33.3	23.6	28.0	15.1	24.0	66.748	< 0.01
	Partially sufficient	11.5	58.4	27.4	2.7	58.4		
	Not sufficient	0.0	45.0	45.0	10.0	10.3		
	Can't say	14.3	57.1	28.6	0.0	7.3		
Provision of microscopic examination for malaria conformation	Yes	14.6	51.7	29.7	4.0	39.0	83.006	< 0.01
	No	25.5	17.0	23.5	34.0	12.2		
	Don't know	14.3	54.0	30.6	1.1	48.8		
Perceived quality of services available	Excellent	35.5	32.3	22.5	9.7	16.0	39.538	< 0.01
	Good	14.6	48.5	30.2	6.7	69.3		
	Poor	0.0	60.6	39.4	0.0	8.5		
	Very poor	0.0	75.0	25.0	0.0	6.2		

* *p* value significance at < 0.05.

#### 6.2.3 Theme: Availability of Drugs, Diagnostic Facilities, Perceived Quality of Service

It was noticed through in‐depth interviews and focus group discussions that people are discouraged from seeking health care from public health facilities due to low‐quality services. Utilization rates have positively impacted when facilities are staffed with professionals, accessible, trustworthy, and reasonably priced. Despite this, a significant number of health facilities may still be avoided due to poor acceptability of services due to social, cultural, or religious reasons. The lack of essential drugs, particularly in public health facilities, was also noted, leading to self‐medication or reliance on traditional healers.

“In the past, there was a lack of understanding among people about the seriousness of malaria. However, presently, people are well‐informed about it and promptly seek medical attention for those affected. As soon as someone contracts malaria, people rush them to the hospital to receive treatment from healthcare professionals, resulting in their swift recovery.”

(Teacher 41 yrs/KII).

“You wouldn't see as many patients in the health centers here if there was no free healthcare. Many people come here because of the free healthcare. But most of the time there is a shortage of drugs and diagnostic facilities”

(Male 35 yrs/IDI).

“Belauri municipality possesses a single public health center, it lacks sufficient resources to handle severe illnesses. Patients are often referred to a larger hospital, located a 2‐3 h’ drive away during the dry season and even longer during the rainy season, to reach Dhangadhi hospital”

(FCHV, 40 yrs/KII).

“We do not have microscope in health center and conformation of diseases are mostly done without it”

(Male, 39 yrs FGD).

### 6.3 Multivariate Logistic Regression Analysis Between Health Service Factors and HSB

Table 7 shows that the individuals who live closer to public hospitals were 2.615 times more likely to seek healthcare from modern health facilities compared to those who lived closest to primary healthcare centers (PHCC) and health post (HP) (AOR = 2.615, 95% CI = 1.18–5.798, *p* = 0.059). This association was nearly statistically significant (*p* = 0.059). Individuals who live closest to primary healthcare centers (PHCCs) were 2.139 times more likely to seek healthcare from modern health facilities (AOR = 2.139, 95% CI = 0.827–5.532). The most common means of reaching the public health facility by bicycle was associated with 8.3 times more likelihood of seeking healthcare from modern health facilities (AOR = 8.324, 95% CI = 3.677–18.844, *p* < 0.001).

**Table 7:** Multivariate analysis of access factors and health seeking behavior.

Variables	Category	AOR	95% C.I. for OR		*p* value
			Lower	Upper	
Nearest public health facility from house	Public hospitals	2.615	1.18	5.798	0.059
	PHCC	2.139	0.827	5.532	
	HP	1			
Most common means to reach at public health facility	By foot	1			< 0.001
	Bicycle	8.324	3.677	18.844	
	Motor‐bike	0.262	0.092	0.75	
	Public vehicle	0.68	0.305	1.517	
Usual time to reach nearest public health facility	< 15 min	1			0.017
	15–30 min	0.359	0.164	0.786	
	30–45 min	0.862	0.267	2.786	
	45–60 min	0.234	0.059	0.918	

Similarly, the means of reaching the public health facility by motorbike was associated with a lower likelihood of seeking healthcare (AOR = 0.262, 95% CI = 0.092–0.75, *p* < 0.001) from modern health facilities, and this association was statistically significant (*p* < 0.05). The usual time it takes to reach the nearest public health facility did not significantly impact the likelihood of seeking healthcare from modern health facilities. The individuals who took more than 15 min to reach the nearest public health facilities were less likely to seek healthcare (AOR = 0.234, 95% CI = 0.059‐0.918, *p* = 0.017) from modern health facilities (Table 7).

The results of Table 8 showed that having to pay for transportation is associated with a lower likelihood of seeking modern health care (COR = 0.275, 95% CI = 0.164–0.461, *p* < 0.001). Additionally, individuals whose usual travel cost to reach the nearest public health facility was less than 60 rupees were also less likely to seek healthcare from modern health facilities (COR = 0.744, 95% CI = 0.640–0.865, *p* < 0.001).

**Table 8:** Multivariate analysis of travel cost and health seeking behavior.

Variables	Category	COR	95% C.I. for OR		*p* value
			Lower	Upper	
Have to pay for transportation	Yes	0.275	0.164	0.461	< 0.001
	No	1			
Usual travel cost to reach nearest public health facility	< 60 rupees	0.744	0.640	0.865	< 0.001
	60 and above rupees	1			

The results given in Table 9 also show that the factors like regular availability of service providers (AOR = 0.155, 95% CI = 0.054–0.442, *p* < 0.001), most of the time availability of service provider (AOR = 0.115, 95% CI = 0.039‐0.334, *p* < 0.001) and convenient opening hours of the public health facility were negatively associated with health seeking behavior (AOR = 0.301, 95% CI = 0.156–0.581, *p* < 0.001) from modern health facility. However, the perceived skill of service providers to treat malaria (AOR = 0.158, 95% CI =, *p* = 2.772) and getting sufficient time to make complaints with service providers (AOR = 0.023, 95% CI = 0.474–0.249, *p* = 0.902) were not significantly associated with health seeking from modern health facility.

**Table 9:** Multivariate analysis of health facility related factors and health seeking behavior.

Variables	Category	AOR	95% C.I. for OR		*p* value
			Lower	Upper	
Availability of service providers in nearest public HF	Regular available	0.155	0.054	0.442	< 0.001
	Most of time available	0.115	0.039	0.334	
	Most of the time unavailable	1			
Perceived skill of service provider to treat malaria in nearest public HF	Skilled	0.158	1.533	0.847	2.772
	Not skilled	1			
Get sufficient time to put complaints with service providers	Yes	0.023	0.474	0.249	0.902
	No	1			
Opening hours of public HF	Convenience	0.301	0.156	0.581	< 0.001
	Not convenience	1			

Table 10 shows that both factors shorter waiting time for consultation (COR = 0.106, 95% CI = 1.931–0.869, *p* = 4.289) and difficulty to understand advice from service provider (COR = 0.000, 95% CI = 3.009–1.79, *p* = 5.057) were not significantly associated with seeking health care from modern health facility.

**Table 10:** Multivariate analysis of health facility characteristics and health seeking behavior.

Variables	Category	COR	95% C.I. for OR		*p* value
			Lower	Upper	
Waiting time (in minute) for consultation after registration in public HF	< 15 min	0.106	1.931	0.869	4.289
	> 15 min	1			
Across with difficulty to understand the advices/words of service provider	Yes	0.000	3.009	1.79	5.057
	No	1			

The results of Table 11 also indicate that the availability of sufficient drugs was found to have an inverse association with health‐seeking behavior (AOR = 0.55, 95% CI = 0.244–1.24, *p* = 0.149) from modern health facilities. This relationship was not considered statistically significant, as the p‐value of 0.149 is greater than 0.05. The provision of microscopic examination for malaria confirmation was found to have a positive association with health‐seeking behavior (AOR = 1.668, 95% CI = 0.97–2.87, *p* = 0.065) to modern health facilities, but this relationship was also not considered statistically significant due to a p‐value of 0.065 greater than 0.05. On the other hand, the perceived quality of services available was found to have a significant association with health‐seeking behavior to modern health facility but perceived the services to be good having a lower likelihood of seeking care (AOR = 0.256, 95% CI = 0.154–0.424, *p* < 0.001) from modern health facility. This relationship was considered statistically significant because the *p*‐value of 0.000 was less than 0.05 (Table 11).

**Table 11:** Multivariate analysis of availability of commodities, facilities, perceived quality of service and health seeking behavior.

Variables	Category	AOR	95% C.I. for OR		*p* value
			Lower	Upper	
Sufficiency of drugs	Sufficient	0.55	0.244	1.24	0.149
	Not sufficient	1			
Provision of microscopic examination for malaria conformation	Yes	1.668	0.97	2.87	0.065
	No	1			
Perceived quality of services available	Good	0.256	0.154	0.424	< 0.001
	Poor	1			

## 7 Discussion

The focus of malaria control and prevention efforts is to reduce mortality and infection rates through effective case management and vector control measures such as promotion of appropriate health‐seeking behavior, use of insecticide‐treated bed nets and mosquito elimination [22, 23]. Lack of widespread adoption of seeking correct health care from appropriate health facilities, and the use of preventive measures among the affected population, is the key obstacle in controlling malaria, making community involvement in malaria prevention is essential for successful control programs. This study has been conducted to assess health service‐related factors affecting health‐seeking behavior during suspected malaria fever in risk area of Nepal. Findings of this study will be helpful to strengthen the malaria control programme and support for policy makers, planners, managers, learners and other interested people involved in malaria control and elimination activities. The current study has assessed the influence of various health service factors on health‐seeking behavior during suspected malaria fever, including the proximity of a health facility, mode of transportation, travel cost, waiting time at the facility, the availability and perceived competency of service providers in treating malaria, the availability of drugs and facilities, and the communication and behavior of the providers.

In this regard, the proximity of health facilities has been established as a significant predictor of seeking care from modern health facilities in multiple studies. In Burkina Faso, proximity to a health facility was associated with a 1.5‐fold increase in the odds of seeking care (AOR = 1.5, 95% CI 1.2–2.10; *p* = 0.01) [24] and those living within 3.5 km of a health facility were 6.5 times more likely to seek care compared to those living further away (AOR = 6.5; CI = 1.74–24.25) in Laos [25]. Similarly, in Ghana, it also established that those who took 1 to 2 h to get to a health facility were 5 times more likely to visit a health facility (AOR = 5.29; 95% CI: 1.97–1,427; *p* = 0.001) [26].

In addition, the choice of healthcare provider was significantly affected by increased travel time, and a rise in travel time by 1 h leads to a 12% decrease in the probability of seeking healthcare in Ghana [27]. Several other studies have also shown that the distance to a health facility and mode of transportation influence healthcare‐seeking behavior, particularly among women [28, 29, 30]. In this context, the current study also supports these findings and found that proximity to a health facility was positively associated with seeking modern healthcare.

Nevertheless, use of a motorbike (AOR = 0.262, 95% CI = 0.092–0.75, *p* < 0.001) and a travel time of more than 15 min to reach the facility (AOR = 0.234, 95% CI = 0.059–0.918, *p* = 0.017) were negatively associated with seeking modern healthcare. The findings of the current study align with the majority of previous research, but contradict the conclusion from China [31], who found that proximity to health facilities did not impact the decision to seek treatment from modern healthcare. Previous studies have consistently shown the importance of transport costs in determining an individual's decision to seek medical treatment during an illness. The research from Brazil demonstrated that transportation expenses posed a hindrance to accessing treatment in Brazil [32]. This finding was supported study from Gambia, which indicated that 54% of survey participants who visited a healthcare facility had to cover transportation costs out of their own pockets, with a median expense of £1.07 [33]. Another study further reinforced this idea, revealing a strong relationship between transport costs and treatment‐seeking behavior in British Columbia [34]. However, a study from Ghana found that travel costs did not significantly affect health‐seeking behavior [27], and another study also reported a negative relationship between travel costs and health‐seeking behavior [35].

The results of the current study also showed a significant association between travel costs and health‐seeking behavior (*p* < 0.05). The study found that paying for transportation reduced the likelihood of seeking modern health care (COR = 0.275, 95% CI = 0.164–0.461, *p* < 0.001) and lower travel costs reduced the likelihood of seeking care from modern facilities (COR = 0.744, 95% CI = 0.640–0.865, *p* < 0.001). These results are consistent with previous studies and suggest that proximity to health facilities and transportation costs impact healthcare‐seeking behavior. Close proximity and lower costs increase the likelihood of seeking care, while distance and paying for transportation decrease it. Regarding other health facility‐related factors affecting health‐seeking behavior, the current study results and previous studies concur on the impact of various health facility‐related factors on health‐seeking behavior. According to the current study results, the regular availability of the service provider and most of the time availability of service provider, as well as convenient opening hours, were found to have a significant negative effect on health‐seeking behavior. The findings align with prior research, indicating that the presence or lack of staff and resources significantly impacts the decisions made regarding seeking healthcare [36, 37]. This highlights the significance of ensuring adequate personnel and equipment to influence positive health‐seeking behavior.

The current study also expands on these findings by showing that regular availability and convenience of opening hours, in particular, are important factors for seeking care from modern health facilities. In contrast, the perceived skill of service providers to treat malaria and getting sufficient time to put complaints with service providers were not found to be significant factors affecting health‐seeking behavior. Contrary to previous research from Nigeria found that staffing adequacy and drug availability were crucial in shaping health‐seeking behavior, the current study highlights a different aspect [38]. Shorter waiting time for consultation and difficulty to understand advice from service providers were also not found to be significant factors affecting health‐seeking behavior in the current study. This contrasts with the results from Ghana [27], showed that increased waiting time has a substantial impact on a person's selection of healthcare provider.

Furthermore, friendly behavior from service providers did not have a significant impact on health‐seeking behavior in the current study, contrary to the findings of FitzGerald and Hurst, 2017 that showed a significant positive relationship between bias among healthcare professionals and health‐seeking behavior [39]. Sufficiency of drugs in health facilities and provision of microscopic examination for malaria confirmation were also not found to be significant factors affecting health‐seeking behavior in the current study. This differs from previous studies from Mozambique and Kenya that showed the importance of availability of staff and equipment [36, 37]. Finally, the current study found that the perceived quality of services available had a significant impact on seeking care, with those perceiving good services having a lower likelihood of seeking care from modern health facilities. This supports previous findings from Nigeria, that the quality of service delivery at the health facility affects health‐seeking behavior [38]. The current study provides new insights into the specific factors that influence health‐seeking behavior in the context of seeking care from modern health facilities and supports the results of previous studies on the importance of availability of staff and equipment, quality of service delivery, and waiting time in determining health‐seeking behavior.

### 7.1 Limitation of the Present Study

The results of the current study, conducted in the Kanchanpur district of Nepal, differ from previous studies due to variations in research design, participant characteristics, and sampling methods. This study employed a mixed‐method design, including a cross‐sectional survey and qualitative components, to gather data on health‐seeking behavior during suspected malaria fever. While the mixed‐method approach provides a nuanced understanding, it is important to note that causality cannot be established through this study design. As being a cross‐sectional study, this study employs an observational design and data were collected from a population at a single point in time. Consequently, it can only identify associations rather than establish cause‐and‐effect relationships. It does not account for the sequence of events, making it unclear whether an exposure preceded the outcome or vice versa. Additionally, this study may fail to control for confounding variables—factors that influence both the exposure and the outcome, and also susceptible to recall bias, as respondents may forget or misreport past behaviors. Furthermore, self‐reported data may be influenced by social desirability bias, leading participants to provide responses they believe are more acceptable rather than entirely accurate. However, results of this cross sectional study are valuable for identifying associations, and generating hypotheses for further research, they have notable limitations in establishing causality, addressing rare outcomes, and managing confounding factors. So, researchers have carefully considered these drawbacks when designing studies and interpreting results. Future research could benefit from different study designs and methods to better understand the factors that contribute to health‐seeking behavior during malaria fever.

## 8 Conclusion

Means of reaching the public health facility, time required to reach the facility, transportation expenses, travel costs, availability of service providers, opening hours of the health facility, and perceived quality of service play crucial roles in shaping individuals’ decisions to seek healthcare for malaria fever. So the plan, strategies and action of the Nepal Government should be focus on improving affordability to ensure that individuals can reach healthcare facilities conveniently and without financial strain, establishing additional facilities in underserved areas or using mobile clinics to decrease travel time to health facilities, transportation subsidies or fee waivers, to mitigate the financial barriers that hinder individuals from seeking healthcare, recruitment and retention of healthcare professionals to ensure consistent and regular availability of service providers, extend the opening hours of health facilities to accommodate the diverse schedules of individuals, making it easier for them to seek healthcare when need, implement quality improvement programs to enhance the perceived quality of healthcare services, focusing on patient satisfaction and effective treatment, and launch public health campaigns to raise awareness about the importance of timely healthcare seeking for malaria fever.

## Author Contributions

**Ashok Kumar Paudel:** conceptualization, writing – original draft, methodology, formal analysis, writing – review and editing, data curation, supervision. **Muni Raj Chhetri:** investigation, validation, writing – review and editing, supervision, data curation, writing – original draft, methodology, conceptualization. **Prem Prasad Panta:** validation, data curation, software, formal analysis, writing – review and editing, supervision. **Nibha Rani Pandey:** methodology, validation, visualization, formal analysis, supervision, writing – review and editing, conceptualization, writing – original draft.

## Ethics Statement

Ethical Approval was obtained from Nepal Health Research Council/Nepal (March 3, 2023/Ref no.‐2041) and Written Informed Consent was obtained from respondents. Respondent confidentiality was safeguarded through the use of anonymity for all documents containing the information they supplied.

## Conflicts of Interest

The authors declare no conflicts of interest.

## Transparency Statement

The lead author Ashok Kumar Paudel affirms that this manuscript is an honest, accurate, and transparent account of the study being reported; that no important aspects of the study have been omitted; and that any discrepancies from the study as planned (and, if relevant, registered) have been explained.

## Data Availability

The data that support the findings of this study are available from the corresponding author upon reasonable request.
